# Modelling homeostatic control of high-frequency post-synaptic transmission and its effect on metabolic efficiency in the auditory brainstem

**DOI:** 10.1186/1471-2202-14-S1-P167

**Published:** 2013-07-08

**Authors:** Yann Sweeney, Jeanette Hellgren-Kotaleski, Matthias Hennig

**Affiliations:** 1 IANC, School of Informatics, University of Edinburgh, UK; 2 Department of Computational Biology, KTH, Stockholm, Sweden

## 

Intrinsic electrical properties of neurons are controlled by a number of homeostatic mechanisms, among which is the modulation of conductances of voltage-dependent ion channels. One such example is mediated by Nitric Oxide (NO) in principal neurons of the Medial Nucleus of the Trapezoid Body (MNTB) in the auditory brainstem. These act as relay neurons, receiving excitatory input and transmitting inhibitory signals to the auditory nuclei involved in sound localisation. NO is released here in an activity-dependent manner and switches the basis of action potential (AP) repolarisation from K_v_3 to K_v_2, decreasing intrinsic excitability and improving faithful following of high frequency input trains [1]. We have replicated these effects in a biophysically detailed neuron model and have measured both transmission fidelity and metabolic efficiency of AP generation, quantified by the Na^+^/K^+ ^charge overlap ratio [3], across varying states of NO activation.

**Figure 1:**
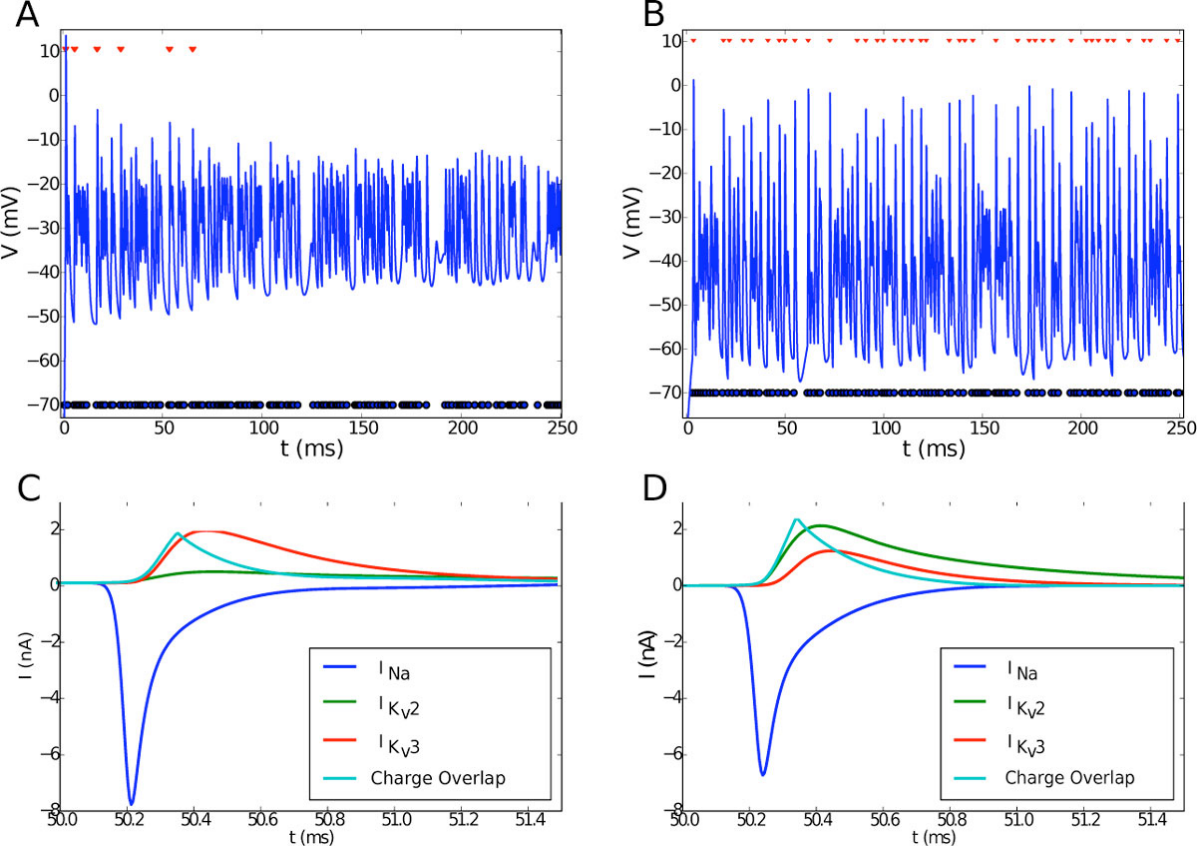
**The NO-mediated conductance state (B) improves transmission ratios at 800 Hz compared to the naïve state (A)**. Blue circles represent Poisson synaptic input and red triangles represent a successfully evoked AP. The lower panels show the Nav, K_v_2 and K_v_3 currents during an AP, with the charge overlap in cyan. The area under the cyan curve is used as a measure of AP metabolic efficiency [3] and is higher in the NO-mediated case **(D) **compared to the naïve **(C)**, thus rendering it less efficient.

It is observed that increasing K_v_2 conductance leads to improved post-synaptic transmission at high frequencies, while also decreasing the metabolic efficiency of an action potential. The location of K_v_2 channels adjacent to Na_v _channels at the axon initial segment (AIS), as opposed to K_v_3, which is located at the soma, is found to be crucial in determining how it affects metabolic efficiency. Figure 1 illustrates that NO mediates transition between a metabolically efficient state sufficient to perform its function at low activity and a metabolically inefficient state required in order to sustain transmission fidelity at high frequencies. This finding provides a plausible justification for the presence of an activity-dependent switch of dominant potassium channel in the MNTB. This effect has subsequently been observed in a CA3 pyramidal cell model [3], a more generic neuron morphology in which NO is also known to act on K_v _conductances [1].
